# A Comparative Study of Common Anesthetics Propofol, Sevoflurane, Isoflurane and Ketamine on Lipid Membrane Fluidity

**DOI:** 10.3390/ijms26031337

**Published:** 2025-02-05

**Authors:** Muhammad Bilal Siddique, Ehsan Nozohouri, Yeseul Ahn, Sumaih Zoubi, Ulrich Bickel, Juyang Huang

**Affiliations:** 1 Department of Physics and Astronomy, Texas Tech University, Lubbock, TX 79409, USA; bilal.siddique@ttu.edu; 2 Department of Pharmaceutical Sciences, School of Pharmacy, Texas Tech University Health Sciences Center, Amarillo, TX 79106, USAulrich.bickel@ttuhsc.edu (U.B.); 3 Center for Blood-Brain Barrier Research, School of Pharmacy, Texas Tech University Health Sciences Center, Amarillo, TX 79106, USA

**Keywords:** endothelial membrane, fluorescence anisotropy, RBC, permeability, dipyrene-PC, DPH-PC

## Abstract

The membrane fluidity increases induced by popular anesthetic agents (propofol, isoflurane, sevoflurane, and ketamine/xylazine) were measured at the clinical and supra-clinical concentrations in red blood cell (RBC) membrane as well as four model membranes. Membrane fluidity changes were monitored using the excimer/monomer (E/M) ratio of dipyrene-PC and fluorescence anisotropies of DPH-PC and TMA-DPH. Propofol, sevoflurane and isoflurane increased membrane fluidity instantaneously. The largest increase occurs in membranes made of saturated lipids. RBCs were labeled with TMA-DPH, and the increase in membrane fluidity at clinical concentrations of isoflurane and sevoflurane was more than that induced by ten times the legal limit of alcohol in human blood. However, membrane fluidity was essentially unchanged by ketamine/xylazine up to 210 µM. These results strongly correlate with our recent in vivo experiments and reveal a clear connection between increasing membrane fluidity in model membranes, increasing the blood–brain barrier (BBB) permeability in mice, and inducing effective anesthesia in animals. Interestingly, at the most commonly used clinical concentrations, the membrane fluidity increases induced by propofol, sevoflurane, and isoflurane were very similar, despite the fact that different categories of anesthetics were used and their chemical concentrations were different by 100 times. This indicates that at clinical concentrations of these anesthetics, a similar level of membrane disruption at the BBB is achieved. Thus, our results strongly support the lipid hypothesis of the mechanism of general anesthetics.

## 1. Introduction

Anesthetic agents and sedative drugs are widely used to enable medical procedures and surgeries. The detailed molecular mechanisms and the long-term neurotoxic effects of anesthetics have been extensively investigated. Currently, there are competing hypotheses about the molecular mechanisms of general anesthetics. The well-known Meyer–Overton correlation, which links the potency of general anesthetic molecules to their olive oil/gas partition coefficients, led to the lipid hypothesis—anesthetic agents have high lipid solubilities and induce anesthesia by disrupting the normal functioning of neural membranes and affecting membrane proteins through some lipid-mediated processes [1,2]. It has been shown that anesthetic agents, such as isoflurane, sevoflurane and propofol, can decrease the gel-to-fluid phase transition temperature of the DPPC lipid bilayer and alter the domain structure of lipid membranes [3,4,5,6]. However, some scientists believe that general anesthetics at clinical concentrations do not produce sufficient membrane changes to affect ion channel functions [7]. On the other hand, the protein hypothesis suggests that anesthetic agents bind to active sites of ion channels or receptors in the central nervous system (CNS) directly and inhibit or enhance the functions of these proteins [8,9,10,11].

Certain drug combinations have been employed as intravenous anesthetics, such as ketamine/xylazine at the 10/1 *w*/*w* ratio [12]. Ketamine is a sedative and analgesic with simple administrative characteristics and also has a great ability to combine with other drugs; in addition, xylazine is being used because of its sedative and analgesic properties [13]. Propofol is another injectable drug that is utilized to initiate and control anesthesia [14]. Propofol has been studied for decades but its mechanisms are still not well understood. It is assumed that propofol and other anesthetic drugs have unique connections with receptors [15]. It has also been suggested that propofol requires various binding sites that γ-aminobutyric acid (GABA), benzodiazepines and barbiturates are using as well [16].

**Figure 1:**
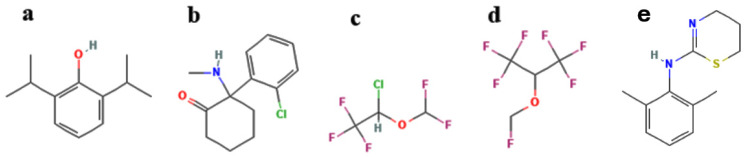
Chemical structures of (**a**) propofol, (**b**) ketamine, (**c**) isoflurane, (**d**) sevoflurane, and (**e**) xylazine.

There are a large number of anesthetics available that differ in their delivery methods (inhaled or intravenous) and in their clinical concentrations, ranging from micromolar to millimolar. Based on the lipid hypothesis, in order to produce the anesthetic effects, certain level of membrane disruption has to be achieved at the clinical concentrations of anesthetic agents. Thus, a good way to test the hypothesis is to compare the effects of different anesthetic agents on well-defined lipid membranes at their clinical concentrations. In this study, we compare the increases in membrane fluidity due to clinical and supra-clinical concentrations of four anesthetic agents (see Figure 1): two intravenous anesthetics (ketamine/xylazine and propofol) and two inhalation volatile anesthetics (sevoflurane and isoflurane). Previously, we have shown that isoflurane at the clinical concentration of 1 mM increases the membrane fluidity in several types of model membranes [17]. Furthermore, our in vivo experiments showed that isoflurane and sevoflurane, but not ketamine/xylazine, increase the blood–brain barrier (BBB) permeability in mice [18]. Propofol is a widely used intravenous anesthetic, and it is believed to interact with GABA-mediated chloride channels in the brain and amplify the inhibitory effects of this neurotransmitter. Very recently, we found that propofol increases BBB permeability to sucrose in mice [19], and others reported enhanced permeability in a human stem cell-derived BBB model [20].

There were two common weaknesses in many published studies. First, due to the fact that membrane disruptions are usually small at the clinical level, the concentrations of anesthetics used were often far above the clinical concentrations. Thus, the clinical relevance of the conclusions can be questionable. Second, the biochemical phase state of the membranes was often uncertain. Since biomembranes are quite diverse, understanding the anesthetic effects on various types of membranes is critically important. Our study examines the effects of the anesthetics on four different types of model membranes, pure DPPC (gel-phase), POPC/Chol (liquid-disordered phase), BSM/DOPC/Chol (liquid-ordered phase or lipid rafts), and a five-lipid mixture that mimics brain endothelial cell membrane. Furthermore, the effects of the anesthetics were also tested with washed human red blood cells (RBCs). All model membranes were labeled with dipyrene-PC and DPH-PC fluorescent probes separately, and the membrane fluidity changes were monitored by the excimer/monomer (E/M) ratio of dipyrene-PC and steady-state fluorescence anisotropy of DPH-PC before and after adding the anesthetic agents. The RBCs were labeled with a TMA-DPH fluorescent probe, and the changes in the anisotropy value were measured. The measured membrane fluidity changes were also compared with those produced by high levels of alcohol, which has been studied extensively [21].

The measured fluorescence anisotropy and dipyrene-PC E/M values can have small variations, depending on the sample preparation, instrument adjustment, and the chemicals used. For a comparative study, it is essential to measure all samples using the same protocol and under the same experimental conditions. Thus, it was necessary to repeat some isoflurane measurements in our earlier study [17] in order to make a fair comparison. Furthermore, the POPC/Chol samples in the earlier study contained 35% cholesterol. In this study, the composition has been changed to POPC/Chol = 90/10, which gives a better representation of a liquid-disordered phase.

## 2. Results and Discussion

### 2.1. Dipyrene-PC E/M Ratio

**Figure 2:**
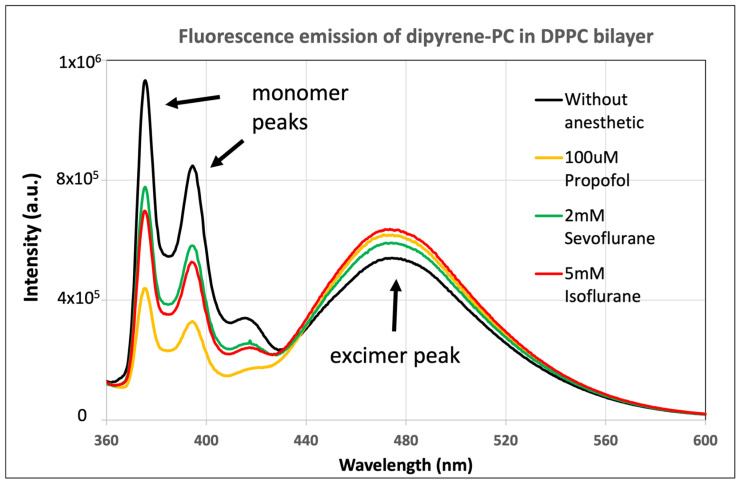
Fluorescence emission spectra of dipyrene-PC in DPPC lipid bilayer at 37 °C with 100 µM propofol, 2 mM sevoflurane, 5 mM isoflurane, and without anesthetic.

Dipyrene-PC is a unique fluorescent probe that has a PC headgroup, and both acyl chains have a fluorescence pyrene group. Dipyrene-PC has often been used to study the phase states of lipid membranes, including mapping out ternary phase diagrams [22]. The emission spectra of dipyrene-PC in the DPPC lipid bilayer with and without anesthetic drugs are shown in Figure 2. The fluorescence emission from individual pyrene groups is called monomer emission and has two sharp characteristic peaks at 376 nm and 398 nm. An excimer is formed when two nearby pyrenes are joined together, and the emission is marked with a broad peak centered at 470 nm. It can be observed from Figure 2 that the monomer peak intensity decreased and the excimer peak intensity increased with the presence of anesthetic agents. The E/M ratio, which is defined as the ratio of excimer peak intensity at 470 nm to monomer peak intensity at 376 nm, is sensitive to membrane fluidity. When a membrane is more fluid, it leads to the formation of more excimers and a higher E/M ratio.

**Figure 3:**
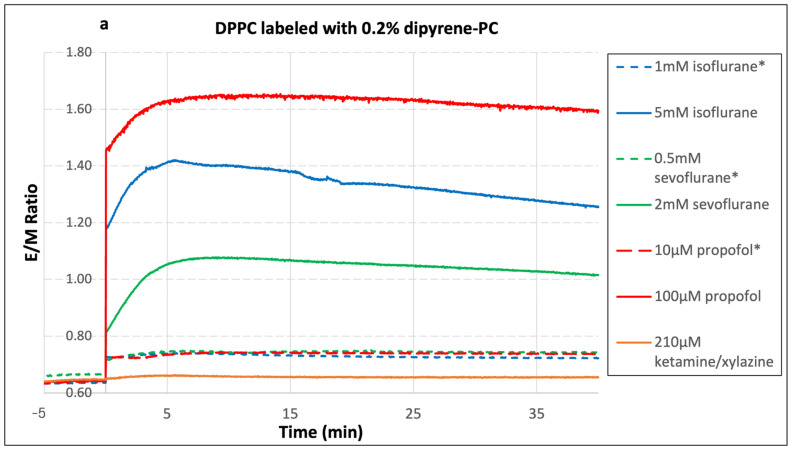

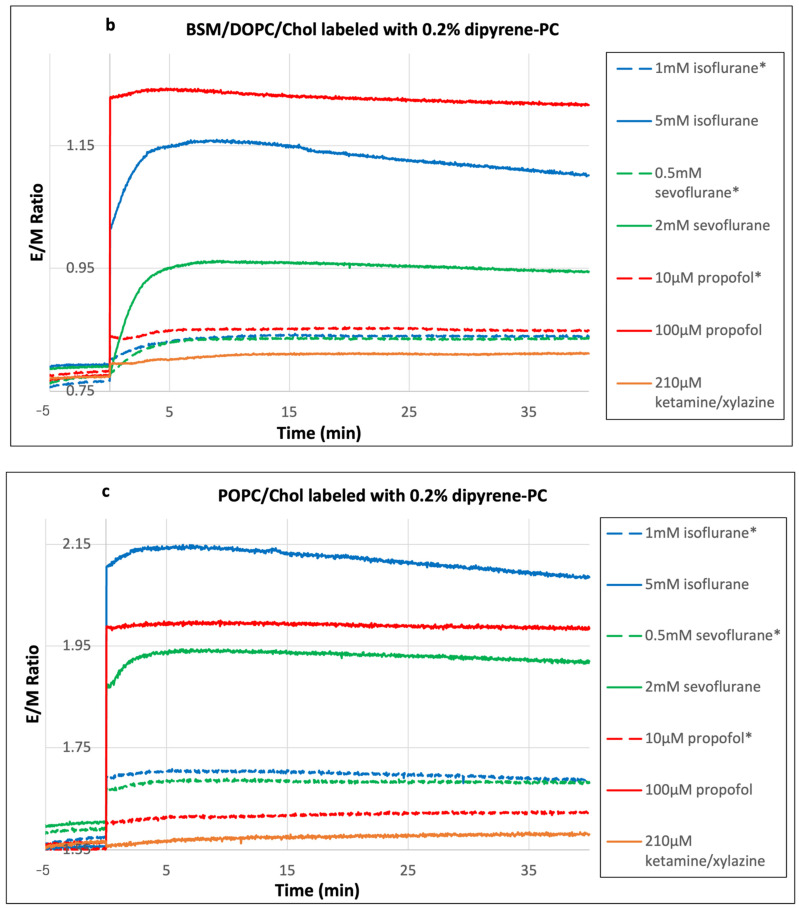

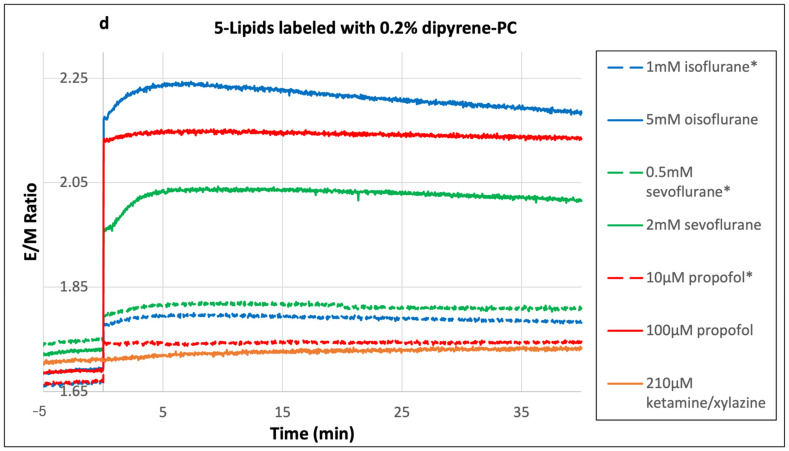
Changes in dipyrene-PC E/M fluorescence intensity ratio at some selected clinical and supra-clinical concentrations of sevoflurane, isoflurane, propofol, and ketamine/xylazine in (**a**) DPPC, (**b**) BSM/DOPC/Chol = 60/5/35, (**c**) POPC/Chol = 9/1, and (**d**) BSM/POPC/POPS/POPE/Chol = 14.5/31/7.4/24.6/22.5 lipid bilayers. Each curve came from an individual measurement. Anesthetic concentrations with an asterisk (*) sign are considered to be clinical concentrations.

Figure 3 shows changes in the dipyrene-PC E/M fluorescence intensity ratio at some selected clinical and supra-clinical concentrations of sevoflurane, isoflurane, propofol, and ketamine/xylazine in the four model membrane systems described earlier. It can be seen in Figure 3 that the E/M ratio increased as soon as anesthetics were added, indicating that the changes in membrane fluidity were immediate. A unique advantage of using dipyrene-PC is that it allows us to monitor sudden changes in membrane fluidity.

The E/M ratio continued to increase slowly in the next 5–7 min, most likely due to more anesthetic reaching the inner bilayers of multi-lamellar vesicles through diffusion. For the time window between 10 and 40 min, the E/M ratio showed gradual decreases for samples with isoflurane and sevoflurane. Since isoflurane and sevoflurane are volatile agents, the likely explanation is that a small quantity of isoflurane and sevoflurane escaped from the sample cuvettes during the measurement. Therefore, we calculated the average E/M ratio between 8 and 15 min for each sample and the complete results are summarized in Table 1. In Table 1 and other figures, the anesthetic concentrations with an asterisk (*) sign are considered to be clinical concentrations. Every experiment listed in Table 1 was repeated at least three times.

Comparing four different lipid bilayers, judging from percentage increases, a pure DPPC bilayer is most affected by the anesthetic agents (see Table 1). The clinical concentrations of 10 µM propofol, 1 mM isoflurane and 0.5 mM sevoflurane have similar effects on the DPPC bilayer with about 15% increase in the E/M ratio, whereas the effects at the clinical concentration of 1 µM–5 µM propofol and 0.25 mM sevoflurane were quite less. Unexpectedly, we tried the clinical concentration of 42 µM ketamine/xylazine multiple times, but no measurable change in the E/M ratio was detected. Furthermore, the supra-clinical concentrations of these drugs produced a larger increase in the E/M ratio. At clinical concentrations of propofol, sevoflurane and isoflurane, the gel/fluid phase transition temperature of DPPC is still above 37 °C, and the data indicate that these anesthetics can induce a more fluid (gel-phase) membrane made of saturated lipids. These results are consistent with other reports that the propofol lowers the phase transition temperature of the saturated DPPC bilayer and helps to fluidize lipid membranes [4].

The second lipid system we investigated was BSM/DOPC/Chol, and its lipid composition was selected from the phase diagram to represent cholesterol-enriched lipid rafts in cell membranes [23]. As expected, the relatively low values of the E/M ratio (~0.77) clearly indicate that this membrane is far more ordered than the POPC/Chol and five-lipid membranes, which have E/M ratios of 1.6 to 1.7. The clinical concentrations of propofol, sevoflurane and isoflurane produced about a 9% increase in the E/M ratio, which is less than the values in the DPPC bilayer. The same trends can be observed for their supra-clinical concentrations as well. These anesthetics affect the fluidity of lipid raft domains that are rich in cholesterol. Finally, POPC/Chol and five-lipid bilayers (that mimic BBB membrane) showed similar increases in the E/M ratio for propofol, sevoflurane and isoflurane. Therefore, the relatively simple POPC/Chol membrane and more complex five-lipid membrane have similar membrane fluidities and responses to anesthetics. Moreover, the effect of propofol on these last two membranes is reduced to half of its values on BSM/DOPC/Chol membrane, but it remains similar to that of isoflurane and sevoflurane. Finally, all bilayer systems were tested with 42 µM ketamine/xylazine as well, but this did not show any measurable effects even at five times its clinical concentration. This indicates that ketamine/xylazine does not affect lipid membranes much, unlike propofol, sevoflurane and isoflurane.

### 2.2. DPH-PC Anisotropy

**Figure 4:**
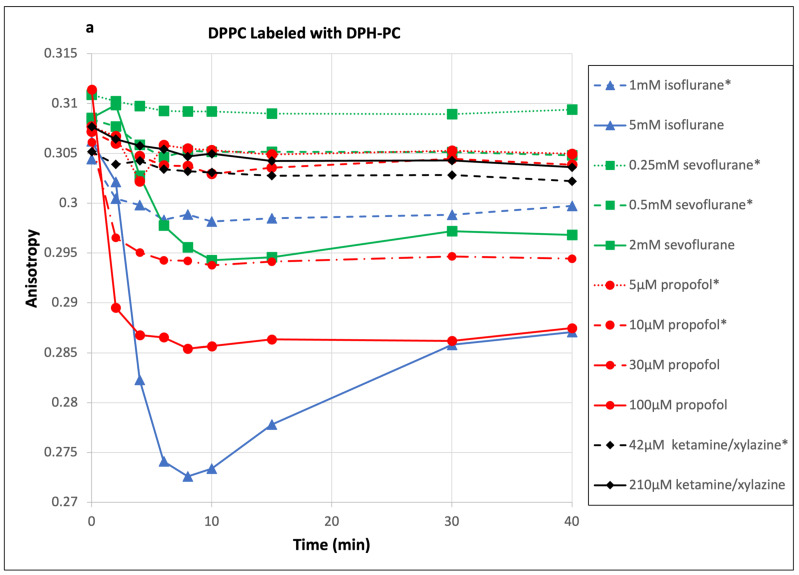

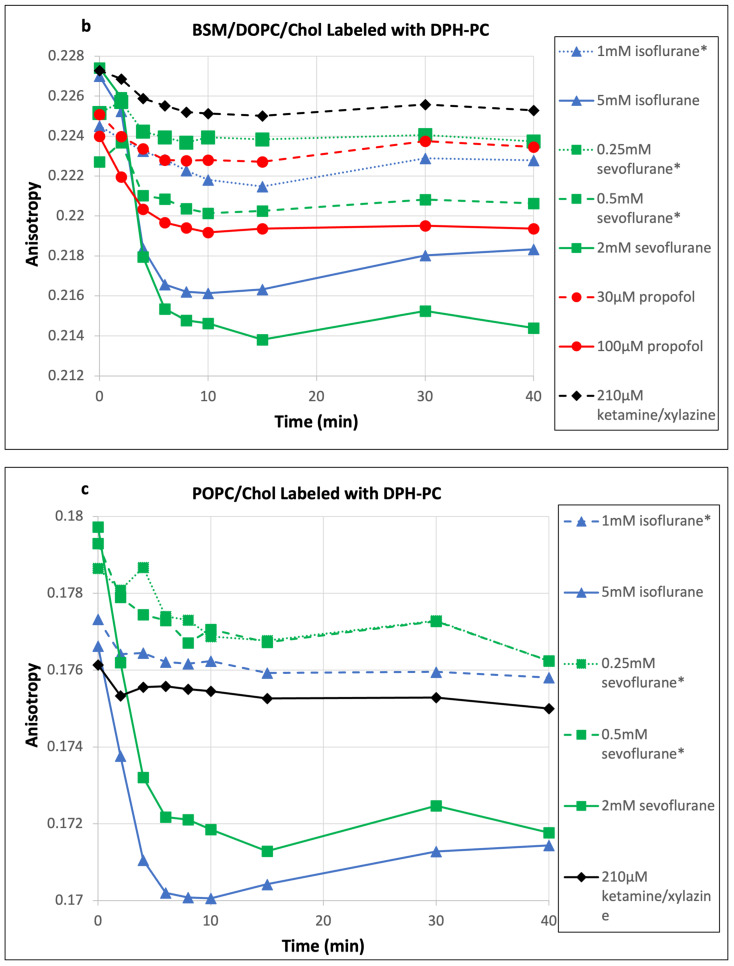

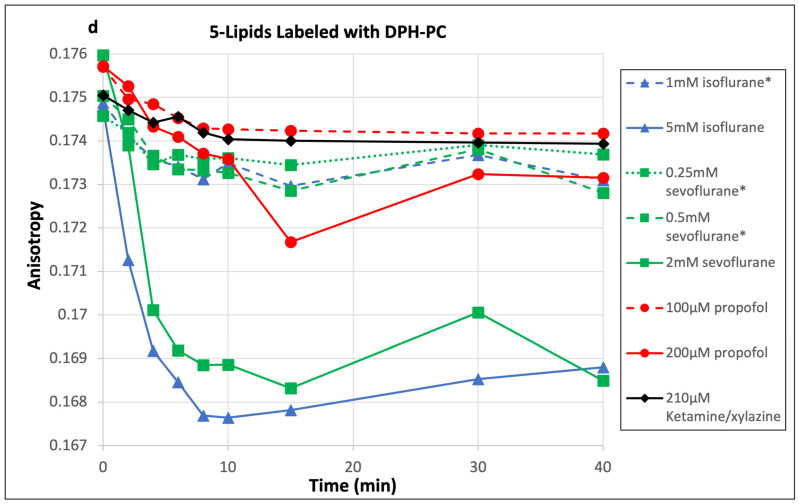
The changes in fluorescence anisotropy of DPH-PC by sevoflurane, isoflurane, propofol, and ketamine/xylazine in (**a**) DPPC, (**b**) BSM/DOPC/Chol = 60/5/35, (**c**) POPC/Chol = 9/1, and (**d**) BSM/POPC/POPS/POPE/Chol = 14.5/31/7.4/24.6/22.5 lipid bilayers. Each curve came from an individual measurement. Anesthetic concentrations with an asterisk (*) sign are considered to be clinical concentrations.

Significant decreases in DPH-PC anisotropy values upon adding anesthetics can be seen in Figure 4. Our experimental setup and adopted methodology (specifically, using RSE sample preparation, T-mode dual-detector measurement, and saturated anesthetic solutions) permit us to put the anesthetics in liposome samples promptly, and the results are reproducible. The fluorescence anisotropy of the DPH-PC fluorescent probe is a standard measurement to detect the changes in membrane fluidity [24]. When a polarized light passes through a sample, it excites those fluorescence probes with dipole moments in the direction of polarized light. The fluorescence lifetime of these molecules is of the order of nanoseconds and the rotation of molecules during the lifetime will change the polarization direction of emission light, which will decrease the anisotropy value. Hence, a decreased anisotropy value reflects a faster rotation of molecules. Therefore, the lipid bilayer is more disordered, and the fluidity of the membrane is increased.

For each sample, DPH-PC anisotropy was measured five times before adding anesthetic. The measured values were quite reproducible with small standard deviations. The anisotropy values dropped during the first 8 min after adding propofol or ketamine/xylazine and then remained relatively stable until 40 min. With sevoflurane and isoflurane, the anisotropy values showed the same decreasing trend for the first 8 min and then started rising slowly. Unlike propofol and ketamine/xylazine, isoflurane and sevoflurane are volatile gas-phase agents, so there is a tendency for these agents to escape from the sample chamber during the measurements. We used the averages of anisotropy values between 8 and 15 min after adding anesthetics to quantify the effects on the membranes. In order to keep the figures readable, only a small number of curves were included in Figure 3 and Figure 4. The data from all experiments are summarized in Table 1 and Table 2. It should be pointed out that unlike the dipyrene-PC E/M ratio, which can be determined within seconds, each anisotropy data point takes two minutes, even with two detectors operating in the T-mode. Therefore, the E/M ratio is much better for capturing a fast change in membrane fluidity than the anisotropy. Interestingly, the maximum E/M ratios for most curves in Figure 3 occur around 8 min after adding anesthetics, while the minimum anisotropy values for most curves in Figure 4 occur around 8–10 min. Thus, both measurements show that membrane fluidity reaches its highest value around 8 min. However, the initial responses of the E/M ratio and anisotropy were very different. The E/M ratio of dipyrene-PC increased sharply after adding anesthetics, while DPH-PC anisotropy decreased more smoothly. The exact cause of the difference is unclear. One possible explanation is that these two fluorescence probes sense fluidity changes in the different regions of the bilayer. The two pyrene groups of a dipyrene-PC are attached to the tenth carbon of the acyl chains. Therefore, the pyrene groups are located very close to the center of the bilayer. On the other hand, the long DPH group of a DPH-PC is attached to the fourth carbon of the acyl chain. Thus, it is reasonable to assume that dipyrene-PC is more sensitive to the fluidity change at the center of the bilayer, and DPH-PC is more sensitive to the fluidity change of the overall acyl chain region. If anesthetic agents cause larger fluidity increases near the center of the bilayers, it would explain the observed difference.

The decrease in the anisotropy value and increase in the E/M ratio both reflect an increase in the fluidity of the bilayer system; hence, both results complement each other. It can be seen in Table 2 that the anisotropy value has the largest percentage decrease in the DPPC bilayer after the anesthetics were added. In agreement, the largest increase in the dipyrene-PC E/M ratio is also in the DPPC bilayer. Since the gel-fluid phase transition temperature of DPPC is 42 °C and all our experiments were carried out at 37 °C, the DPPC bilayer was still in a highly ordered gel state. Thus, the data clearly show that propofol, sevoflurane, and isoflurane are quite effective at fluidizing the tightly packed gel state of saturated DPPC bilayers. These effects can be observed at the clinical concentrations of propofol, sevoflurane and isoflurane. However, the changes in DPH-PC anisotropy by the clinical concentration of ketamine/xylazine (42 µM) were too small to be measured.

Consider the standard deviations of our anisotropy measurement, where the detection limit of anisotropy change is about 0.5%. For some measurements using clinical concentrations of propofol, we found that the changes in DPH-PC anisotropy were too small to be detected by this technique (Table 2). However, the corresponding dipyrene-PC E/M ratio data clearly show that the changes in membrane fluidity by the clinical concentrations of propofol are small but definitely measurable (see Table 1).

Compared to DPH-PC anisotropy, the dipyrene-PC E/M ratio method is faster and more sensitive. The fluorescence anisotropy value of a probe in a membrane not only depends on its rotational diffusion but also on the fluorescent lifetime of the probe. If the fluorescent lifetime becomes shorter, then the probe molecules would have less time to rotate before the emission and that would increase the anisotropy value. The lifetime of fluorescent probes is sensitive to local biophysical environments, such as membrane fluidity and temperature. The fluorescent lifetime of DPH-PC and TMA-DPH is 4–9 ns. Kalb et al. found that the lifetime of DPH in a POPC bilayer becomes longer as cholesterol is added to the bilayer, which is known to increase the membrane order [25]. Barrow and Lentz reported that the lifetime of DPH probes in the DPPC lipid bilayer becomes shorter as DPPC does through a phase transition from the gel phase to the fluid phase [26]. Thus, as membrane fluidity increases, the observed decrease in DPH-PC anisotropy is caused by the faster rotational motion of DPH probes, which has to overcome the opposite effect of a shorter fluorescence lifetime. This makes the DPH-PC anisotropy method less sensitive. On the other hand, dipyrene-PC has an unusually long lifetime of ~100 ns [27]. Thus, the dipyrene-PC E/M ratio is far less sensitive to changes in fluorescence lifetime and is a better measurement of membrane fluidity.

### 2.3. Fluorescence Anisotropy of Red Blood Cells

**Figure 5:**
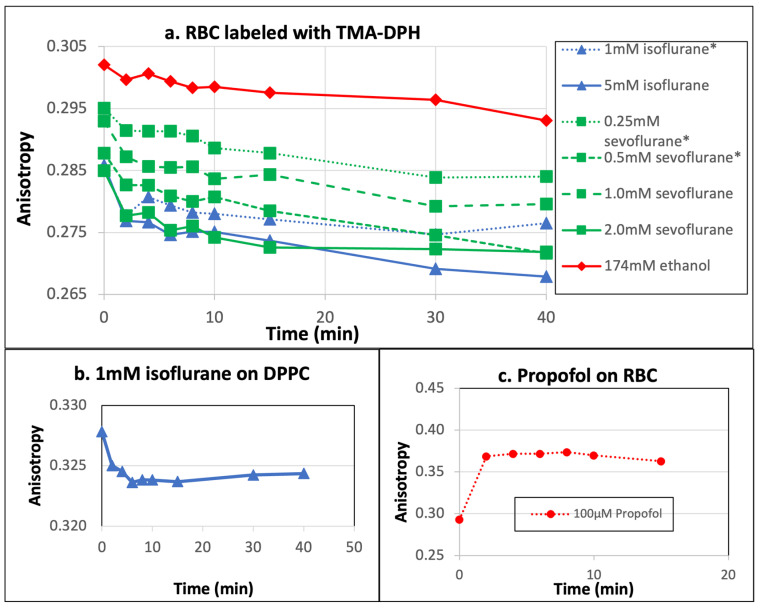
The changes in TMA-DPH fluorescence anisotropy with (**a**) various concentrations of isoflurane and sevoflurane on RBC membranes, (**b**) 1 mM isoflurane on DPPC bilayers, and (**c**) 100 µM propofol on RBC membranes. Each curve came from an individual measurement. Anesthetic concentrations with an asterisk (*) sign are considered to be clinical concentrations.

In addition to the four types of model membranes, we also measured the effects of anesthetics on the fluidity of RBC membranes. Since dipyrene-PC and DPH-PC fluorescent probes cannot be used to label RBC, TMA-DPH was used. It should be pointed out that TMA-DPH only labels the outer leaflet of RBC membranes, so interactions between the probe and interior components of RBC should be negligible. It can be seen in Figure 5a that isoflurane and sevoflurane decrease the anisotropy of TMA-DPH and increase membrane fluidity on more complex RBC membranes. We again compared the initial anisotropy value with the average value between 8 and 15 min after adding anesthetics, and the results are summarized in Table 3. Comparing DPH-PC and TMA-DPH anisotropy values in Table 2 and Table 3, it appears that the membrane fluidity of RBC outer membranes is closer to that of the DPPC bilayer than other model membranes. Furthermore, when ketamine/xylazine was used on RBC membranes, the TMA-DPH anisotropy was essentially unchanged. This result is consistent with our measurements of ketamine/xylazine on model membrane systems.

In order to see whether other substances can also produce similar membrane fluidity increases caused by sevoflurane or isoflurane, we added up to 174 mM ethanol (which is about 10 times the legal content of alcohol in human blood) in RBC samples and compared the changes in anisotropy with the clinical concentrations of anesthetics (Figure 5a). It is quite interesting that the change in TMA-DPH anisotropy due to clinical concentrations of isoflurane and sevoflurane is far larger than that caused by 174 mM ethanol.

As a control, we also labeled the DPPC bilayers with DPH-PC and TMA-DPH separately and found that the percentage changes in anisotropy values after adding anesthetics were similar (Figure 5b). Unexpectedly, we found that the TMA-DPH fluorescence anisotropy increased upon adding the propofol to RBC membranes (Figure 5c). Since propofol was found to increase membrane fluidity in all model membrane systems, this unusual result is likely due to some interactions between propofol and RBC membranes. Using the HPLG technique, researchers found that propofol extensively binds to the serum proteins and RBC membranes [28,29]. Recently, the binding of propofol to serum albumin was investigated at the molecular level using a computational method [30]. If the binding of propofol to membrane proteins causes structural changes to the RBC surface and results in a lower binding affinity of TMA-DPH to RBC outer membranes, it would explain the unexpected TMA-DPH fluorescence anisotropy increase in our experiments.

### 2.4. Sevoflurane and Isoflurane

At the clinical concentrations of isoflurane and sevoflurane, our experimental temperature (37 °C) is still below the gel-fluid phase transition temperature of the DPPC bilayer [24]. Therefore, DPPC bilayers are still in the gel phase, and the largest increase in membrane fluidity of the DPPC bilayer upon adding isoflurane and sevoflurane is not due to the phase transition of DPPC bilayers. Moreover, POPC/Chol and the five-lipids mixture are already in the fluid phase, which can be verified from their lower fluorescence anisotropy values and larger E/M ratio compared to the DPPC bilayer before adding the anesthetics. Hence, it can be concluded that the increase in the membrane fluidity was caused by isoflurane and sevoflurane.

### 2.5. Propofol

There is a large range of reported clinical concentrations of propofol in the literature. The plasma concentration of propofol rapidly shoots and reaches to 10–55 µM during the induction of clinical anesthesia [31]. It has also been found that the plasma concentration remains almost constant at the value of 12.6 µM [32]. The propofol in bovine brain cell membranes strengthens the binding of GABA receptors with an EC_50_ value of 18.7 µM [33] and it also induces a GABA-gated Cl-channel above 10 µM concentrations [34]. The same findings were observed for 1–100 µM propofol induction in supra-optic neurons of rats [35]. It is difficult to estimate an in vivo concentration of propofol because propofol binds easily to RBC and plasma proteins [36]. However, its brain concentration is considered comparatively much higher than surrounding tissues because propofol is lipophilic and binds extensively to RBC in blood [37]. The representing in vitro propofol concentration has been reported from 3 µM to 50 µM [38,39]. In this study, we considered 10 µM or less as the clinical concentration.

Propofol also reduces the phase transition temperature of the DPPC bilayer at high concentrations (>50 µM) [4]. Most likely, at 10 µM of propofol, the DPPC bilayer is still in the gel phase at 37 °C. The human brain lipid membranes consist of both saturated and unsaturated fatty acid phospholipids and have a solid amount of cholesterol as well [40]. The cholesterol-containing POPC membranes and five-lipid membranes also show fluidization. However, the theory of membrane fluidization with the effect of anesthetics has been criticized because the membrane fluidity increase has been observed mostly in in vivo experiments [10] and at supra-clinical anesthesia concentrations [15]. For the DPPC bilayer, the decrease in DPH-PC fluorescent anisotropy is remarkably significant. The other three model membranes are already in the fluid phase at 37 °C, and the effects on membrane fluidity upon adding propofol are not as large. Consequently, the change in DPH-PC fluorescence anisotropy is much less. For RBC, instead of decreasing, the TMA-DPH anisotropy value increases sharply after adding propofol (see Figure 5c). This observation is opposite to its effect on DPH-PC anisotropy in model membranes. The likely explanation is that propofol interacts with some membrane proteins on the RBC membrane and alters the binding of TMA-DPH to RBC. Since propofol increases membrane fluidity in all four model membranes at its clinical concentrations (Table 1), it is reasonable to assume that propofol also increases the fluidity of the RBC membrane, but TMA-PC is not the right probe for this situation. Furthermore, for the protein hypothesis to be valid, there should be specific ion channels or receptors for each anesthetic agent, which is still an ongoing debate.

### 2.6. Ketamine

There are not many reports on the effect of ketamine on the fluidity of model lipid membranes or human RBC membranes. Veilleux-Lemieux et al. studied pharmacokinetics of ketamine/xylazine on rats at the clinical concentration of 42 µM [41]. Using the spin label technique, L Mazzanti et al. observed the change in the fluidity of isolated rat brain synaptic and mitochondrial membranes upon exposing them to supra-clinical concentrations (1–5 mM) of ketamine [42]. Jerabek et al. used simulation modeling and found that ketamine may activate ion channels [43]. In this study, we did not observe detectable changes in DPH-PC anisotropy and the dipyrene E/M ratio at 42 µM clinical concentrations of ketamine/xylazine. However, a small change (up to ~1%) was observed at 210 µM, which is about five times the clinical concentration. Also, no change was observed in TMA-DPH anisotropy for RBC samples even at 210 µM. Thus, at the clinical concentrations, the effect of ketamine/xylazine on membrane fluidity is far less than that of propofol, sevoflurane and isoflurane.

## 3. Materials and Methods

1,2-dipalmitoyl-sn-glycero-3-phosphocholine (DPPC), 1-palmitoyl-2-oleoyl-sn-glycero-3-phosphocholine (POPC), 1,2-oleoyl-sn-glycero-3-phosphocholine (DOPC), 1-palmitoyl-2-oleoyl-sn-glycero-3-phosphoethanolamine (POPE), 1-palmitoyl-2-oleoyl-sn-glycero-3-phospho-L-serine (POPS), and brain sphingomyelin (BSM) were purchased from Avanti Polar Lipids (Alabaster, AL) and cholesterol form Nu Chek Prep (Elysian, MN). 2-(3-(diphenylhexatrienyl)propanoyl)-1-hexadecanoyl-sn-glycero-3-phosphocholine (DPH-PC) and 1,2-bis-(1-pyrenedecanoyl)-sn-glycero-3-phosphocholine (dipyrene-PC) were obtained from Molecular Probes (Eugene, OR). N,N,N-trimethyl-4-(6-phenyl-1,3,5-hexatrien-1-yl)-benzenaminium, 4-methylbenzenesulfonae (TMA-DPH) was purchased from Cayman Chemical (Ann Arbor, MI). The molarity of various phospholipid stocks was determined by phosphate assays [44]. Packed human red blood cells (RBCs) were obtained from Our Blood Institute (Oklahoma City, OK, USA). Propofol, isoflurane, ketamine and xylazine were ordered through Cardinal Health (Dublin, OH, USA) and sevoflurane was purchased from Covetrus North America (Portland ME, USA). Phosphate buffered saline (PBS) of pH 7.4 was prepared by dissolving PBS tablets purchased from Sigma Life Science (St. Louis, MO, USA) in deionized water (~18 MΩ).

### 3.1. Preparation and Labeling of Liposomes

Four types of model membranes were produced: pure DPPC (gel-phase at 37 °C), POPC/Chol = 90/10 (liquid-disordered L_d_ phase), BSM/DOPC/Chol = 60/5/3 (liquid-ordered L_0_ phase) and BSM/POPC/POPS/POPE/Chol = 14.5/31/7.4/24.6/22.5 (that mimics brain endothelial cell membranes) [45]. Each liposome sample was labeled with dipyrene-PC and DPH-PC fluorescent probes separately at the lipid/probe ratio of 500/1. The total lipid concentration of each sample was kept at 100 µM. After lipids and probes were dissolved and mixed in chloroform, the rapid solvent exchange (RSE) method was used to create liposomes one tube at a time [46]. Briefly, the glass test tube was placed on a 50 °C heating block for 1 min. Then, 1.3 mL of PBS buffer was added, and the glass tube was placed on a vortex mixer coupled to a home-built device. The sample was vortexed vigorously, and the pressure was gradually reduced to 3 cm of Hg. This process removes of the majority of solvent, and the remaining traces were removed by vertexing for an additional minute at the same reduced pressure. After that, the sample was sealed under the argon gas and was placed on a mechanical shaker for no less than four days before the measurements. The majority of liposomes created by the RSE method have one or two lipid bilayers ([46] and personal communication). Thus, RSE liposomes have far more outer surface area than typical multilamellar (MLV) liposomes.

### 3.2. Excimer/Monomer (E/M) Ratio Measurements

To measure the E/M ratio of dipyrene-PC, a PTI C61/2000 spectrophotometer with T-mode design in a single photon counting configuration was used. The excitation wavelength was 333 nm, and the slit width of the monochromator was set at 3 nm. The emission intensities of excimer and monomer peaks of dipyrene-PC were collected at 376 nm and 470 nm, respectively, with an 8 nm slit-width for both emission monochromators. The E/M ratio was calculated as E/M = I_470 nm_/I_376 nm_.

### 3.3. Fluorescence Anisotropy Measurements

The steady-state fluorescence anisotropy (*r*) of the DPH-PC fluorescent probe was carried out with the same PTI C61/2000 spectrophotometer. The excitation and emission monochromators were set at 360 nm and 440 nm with a slit width of 6 nm and 8 nm, respectively. The fluorescence anisotropy is defined as:

(1) $$r=\frac{I_{VV}-gI_{VH}}{I_{VV}+2gI_{VH}}\;with\;g=\frac{I_{HV}}{I_{HH}}$$

where $I_{VV}$, $I_{VH}$ are the emission intensities of vertical and horizontal polarized light when the sample was excited with a vertically polarized light, while g is a factor associated with the relative sensitivity of emission channels, which is measured when the excited light polarization was changed to the horizontal direction. Each measurement took 2 min and was carried out on a 4 mL quartz cuvette. A liposome sample was transferred to a cuvette along with a micromagnetic stir bar and was heated to 37 °C before taking the measurement. The total volume of each sample after adding an anesthetic solution was kept at 3 mL. Adding anesthetic solution did not change the probe/lipid ratio, but did dilute the liposomes, particularly at high anesthetic concentrations. However, in the T-mode configuration, both the E/M ratio and anisotropy depend on the ratio of two simultaneously collected fluorescence intensities, so the magnitudes of the intensities reduced by the dilution have a relatively small effect on the calculated values. For example, for the 1 mM (clinical) isoflurane experiment with the DPPC bilayer, the measured E/M ratio increase was 15.73% (Table 1). In the control experiment using the same quantity of buffer without isoflurane, the increase was only 0.15%. For the 5 mM (supra-clinical) isoflurane experiment, the measured increase was 116.8%, and the increase in the control experiment was 2.8%. Therefore, the effect of dilution was quite small.

### 3.4. Washing and Labeling of Red Blood Cells (RBCs)

The initial human RBC stock was at 5 × 10^6^ cells/µL. In total, 0.1 mL of this stock (~5 × 10^8^ RBC) was diluted with 0.9 mL PBS buffer in a plastic centrifuge tube. This 1 mL suspension was centrifuged at 877× *g* for 5 min, which resulted in a lose pellet of RBC at the bottom of the tube. After 0.9 mL supernatant was removed, 0.9 mL of fresh PBS buffer was added. The washing process was repeated four more times to obtain a final 1 mL RBC sample. After several rounds of gentle mixing and dilutions, about 1.5 × 10^6^ RBC was transferred to a quartz cuvette with 3 mL PBS buffer and a magnetic stirrer bar. The total surface area of RBC in our sample was similar to the surface area of a 5 × 10^6^ parasite RBC used in another study [47] since human RBCs are larger in size. Moreover, a 3 mL RSE liposome sample has about 4 × 10^8^ vesicles, which have a similar total surface area as a RBC sample. To label RBC, 40 µL of 26.33 µM TMA-DPH in DMSO was added to the cuvette and incubated at 37 °C for 20 min before taking the anisotropy measurements. The spectrophotometer excitation and emission wavelengths were at 360 nm and 455 nm with the slit widths of 10 nm and 8 nm, respectively.

### 3.5. Measurement Protocol

The saturated solutions of 500 µM propofol and 24.2 mM isoflurane and sevoflurane in PBS buffer were prepared in separate 20 mL glass bottles. Also, the ketamine:xylazine = 10:1 *w*/*w* mixture was prepared at the concentration of ketamine:xylazine = 100 mg/mL:10 mg/mL. All anesthetic solution bottles were tightly sealed with a Teflon cap and paraffin film and were stirred for 12–18 h at room temperature to reach the saturated solutions. Throughout the measurement, the saturated anesthetic solutions were kept at 37 °C. The sample chamber of the spectrophotometer was maintained at 37 °C. The quartz cuvette, containing the liposome sample and a micro stirrer bar, was placed inside that chamber and was given at least 5 min to reach 37 °C before taking the measurements. In order to establish stable baseline values before adding anesthetic agents, the E/M ratio of dipyrene-PC was recorded continuously for 10 min, and the average E/M ratio during the last 5 min was used as the initial values. Fluorescence anisotropy was performed 5 times on DPH-PC-labeled samples, and their average was used as the initial value. The anesthetic agent was added in the cuvette at t = 0, and the E/M ratio of dipyrene-PC labeled samples were collected continuously for 40 min while the first 5 measurements of fluorescence anisotropy (where each measurement took 2 min) for DPH-PC-labeled samples were taken during the first 10 min. Three more anisotropy measurements were gathered at 15, 30 and 40 min. Although the cuvette was capped except for the time duration of adding anesthetic agents, a small amount of isoflurane and sevoflurane must be lost in gas form because of their volatile nature. However, it does not affect our conclusion that isoflurane and sevoflurane increase the membrane fluidity, although the actual effects should be slightly larger. Furthermore, most anesthetic concentrations were selected to match the reported clinical concentrations. The actual binding of anesthetic molecules to lipid membranes depends on their affinities to the membranes, which was not studied here. In general, a low binding affinity to the membrane could result in a high required clinical anesthetic concentration for the same amount of membrane binding.

## 4. Conclusions

In this comparative study, we found that the clinical levels of propofol, sevoflurane and isoflurane increase the membrane fluidity in four types of model membranes, representing highly ordered gel phase, liquid-ordered (raft) phase, liquid-disordered phase, and brain endothelial cell membranes which could contain both liquid-ordered and liquid-disordered domains. Particularly, these anesthetics are quite effective at fluidizing the highly ordered lipid domains made of saturated lipids. In contrast, no detectable membrane fluidity change was found with the clinical levels of ketamine/xylazine. An increase in membrane fluidity indicates a more disordered membrane, which should result in a higher membrane permeability for small molecules. Our results strongly correlate with the recent in vivo experiments in mice. Nozohouri et al. found that BBB permeability to sucrose increased significantly for propofol-infused mice at the clinical plasma concentrations, and stable anesthesia was achieved using ketamine/xylazine followed by a constant IV infusion of propofol [19]. However, it was found in the same study that ketamine/xylazine alone did not have such effects. Furthermore, volatile anesthetics, such as sevoflurane and isoflurane, increase BBB membrane permeability to small molecules and induce anesthesia in mice at clinical concentrations [18,48]. Our results clearly demonstrate that there is a direct correlation between the increase in membrane fluidity in model and natural membranes, the increase in BBB membrane permeability in vivo, and inducing clinical anesthesia in animals at the clinical concentrations of anesthetics used in this study.

A very interesting pattern emerging from our large number of measurements is that at the most commonly used clinical concentrations (i.e., 10 µM propofol, 0.5 mM sevoflurane, and 1 mM isoflurane), the membrane fluidity increases in various membranes by propofol, sevoflurane and isoflurane are very similar (see Table 1 and Table 2), despite the facts that (i) the chemical structures of anesthetics are very different (see Figure 1), (ii) the actual chemical concentrations are different by 100 times, and (iii) sevoflurane and isoflurane are inhalable anesthetics, while propofol is intravenous. Our combined data indicate that similar degrees of BBB membrane perturbations are achieved at the clinical levels of different anesthetics, independent of the type of anesthetics used. In conclusion, our findings strongly support the lipid hypothesis of anesthetics.

## Figures and Tables

**Table 1:** The changes in dipyrene-PC E/M ratio after adding anesthetics. The average measured values during the 5 min period before adding the drug is called “Before adding Drug”. Anesthetic concentrations with asterisk (*) sign are considered to be clinical concentrations. “No change” means that the statistical change is less than the detection limit of 0.5%.

Membranes	Drug Concentrations		Before Adding Drug	Averages Between 8 and 15 min After Adding Anesthetic	Percentage Change
**DPPC**	**Sevoflurane**	0.25 mM *	0.665 ± 0.004	0.710 ± 0.007	6.77%
		0.50 mM *	0.664 ± 0.028	0.744 ± 0.022	12.05%
		**1.0 mM**	0.659 ± 0.030	0.820 ± 0.042	24.43%
		**2.0 mM**	0.642 ± 0.001	1.073 ± 0.029	67.13%
	**Isoflurane**	1 mM *	0.636 ± 0.009	0.736 ± 0.036	15.72%
		**5 mM**	0.643 ± 0.004	1.394 ± 0.049	116.8%
	**Propofol**	1 µM *	0.662 ± 0.003	0.681 ± 0.008	2.87%
		5 µM *	0.669 ± 0.004	0.711 ± 0.006	6.28%
		10 µM *	0.639 ± 0.017	0.742 ± 0.004	16.12%
		**30 µM**	0.656 ± 0.015	0.917 ± 0.014	39.79%
		**100 µM**	0.643 ± 0.027	1.646 ± 0.090	155.99%
		**200 µM**	0.647 ± 0.018	2.184 ± 0.023	237.56%
	**Ketamine/** **xylazine**	42 µM *			No change
		**210 µM**	0.646 ± 0.007	0.658 ± 0.002	1.86%
**BSM/DOPC/Chol**	**Sevoflurane**	0.25 mM *	0.770 ± 0.014	0.811 ± 0.008	5.32%
		0.50 mM *	0.771 ± 0.024	0.835 ± 0.027	8.30%
		**1.0 mM**	0.794 ± 0.009	0.877 ± 0.009	10.45%
		**2.0 mM**	0.789 ± 0.016	0.960 ± 0.024	21.67%
	**Isoflurane**	1 mM *	0.763 ± 0.040	0.839 ± 0.033	9.96%
		**5 mM**	0.793 ± 0.029	1.155 ± 0.034	45.65%
	**Propofol**	1 µM *	0.728 ± 0.011	0.796 ± 0.011	9.34%
		5 µM *	0.780 ± 0.012	0.810 ± 0.007	3.85%
		10 µM *	0.780 ± 0.008	0.851 ± 0.013	9.10%
		**30 µM**	0.767 ± 0.033	0.944 ± 0.018	23.08%
		**100 µM**	0.773 ± 0.004	1.235 ± 0.013	59.77%
		**200 µM**	0.779 ± 0.007	1.499 ± 0.042	92.43%
	**Ketamine/** **xylazine**	42 µM *			No change
		**210 µM**	0.773 ± 0.305	0.809 ± 0.036	4.66%
**POPC/Chol**	**Sevoflurane**	0.25 mM *	1.596 ± 0.016	1.651 ± 0.017	3.45%
		0.50 mM *	1.588 ± 0.008	1.686 ± 0.007	6.17%
		**1.0 mM**	1.586 ± 0.014	1.765 ± 0.008	11.29%
		**2.0 mM**	1.601 ± 0.011	1.939 ± 0.034	21.11%
	**Isoflurane**	1 mM *	1.569 ± 0.010	1.704 ± 0.009	8.60%
		**5 mM**	1.555 ± 0.019	2.139 ± 0.056	37.56%
	**Propofol**	1 µM *	1.596 ± 0.003	1.623 ± 0.004	1.69%
		5 µM *	1.576 ± 0.004	1.609 ± 0.007	2.09%
		10 µM *	1.550 ± 0.007	1.615 ± 0.013	4.19%
		**30 µM**	1.580 ± 0.005	1.763 ± 0.019	11.58%
		**100 µM**	1.563 ± 0.023	1.995 ± 0.032	27.64%
		**200 µM**	1.554 ± 0.010	2.128 ± 0.044	36.94%
	**Ketamine/** **xylazine**	42 µM *			No change
		**210 µM**	1.562 ± 0.008	1.573 ± 0.002	0.70%
**5-Lipids**	**Sevoflurane**	0.25 mM *	1.741 ± 0.012	1.787 ± 0.009	2.64%
		0.50 mM *	1.746 ± 0.022	1.818 ± 0.024	4.12%
		**1.0 mM**	1.713 ± 0.012	1.844 ± 0.013	7.65%
		**2.0 mM**	1.727 ± 0.006	2.037 ± 0.023	17.95%
	**Isoflurane**	1 mM *	1.666 ± 0.053	1.795 ± 0.049	7.74%
		**5 mM**	1.690 ± 0.010	2.232 ± 0.018	32.07%
	**Propofol**	1 µM *	1.708 ± 0.017	1.728 ± 0.009	1.17%
		5 µM *	1.708 ± 0.003	1.734 ± 0.005	1.52%
		10 µM *	1.668 ± 0.012	1.743 ± 0.012	4.49%
		**30 µM**	1.674 ± 0.013	1.859 ± 0.030	11.05%
		**100 µM**	1.689 ± 0.018	2.147 ± 0.018	27.12%
		**200 µM**	1.717 ± 0.021	2.372 ± 0.021	38.15%
	**Ketamine/** **xylazine**	42 µM *			No change
		**210 µM**	1.709 ± 0.0113	1.725 ± 0.003	0.94%

**Table 2:** The decreases in fluorescence anisotropy of DPH-PC after adding anesthetics. The average measured values during the 10 min period before adding anesthetic is called “Before adding anesthetic”. Anesthetic concentrations with an asterisk (*) sign are considered to be clinical concentrations. “No change” means that the statistical change is less than the detection limit of 0.5%.

Membranes	Drug Concentrations		Before Adding Anesthetic	Averages Between 8 to 15 min After Adding Anesthetic	Percentage Change
**DPPC**	**Sevoflurane**	0.25 mM *	0.3109 ± 0.0004	0.3091 ± 0.0002	−0.58%
		0.50 mM *	0.3084 ± 0.0010	0.3052 ± 0.0012	−1.04%
		**1.0 mM**	0.3081 ± 0.0009	0.3017 ± 0.0019	−2.08%
		**2.0 mM**	0.3085 ± 0.0005	0.2948 ± 0.0005	−4.44%
	**Isoflurane**	1 mM *	0.3044 ± 0.0013	0.2985 ± 0.0027	−1.94%
		**5 mM**	0.3062 ± 0.0020	0.2746 ± 0.0034	−10.32%
	**Propofol**	1 µM *			No change
		5 µM *	0.3077 ± 0.0006	0.3052 ± 0.0008	−0.81%
		10 µM *	0.3072 ± 0.0018	0.3034 ± 0.0029	−1.24%
		**30 µM**	0.3072 ± 0.0021	0.2940 ± 0.0034	−4.30%
		**100 µM**	0.3056 ± 0.0001	0.2858 ± 0.0014	−6.48%
		**200 µM**	0.3096 ± 0.0012	0.2325 ± 0.0030	−24.90%
	**Ketamine/** **xylazine**	42 µM *	0.3052 ± 0.0029	0.3030 ± 0.0028	−0.72%
		**210 µM**	0.3077 ± 0.0003	0.3046 ± 0.0018	−1.01%
**BSM/DOPC/Chol**	**Sevoflurane**	0.25 mM *	0.2251 ± 0.0017	0.2238 ± 0.0020	−0.58%
		0.50 mM *	0.2227 ± 0.0012	0.2202 ± 0.0016	−1.12%
		**1.0 mM**	0.2252 ± 0.0017	0.2191 ± 0.0005	−2.71%
		**2.0 mM**	0.2274 ± 0.0014	0.2144 ± 0.0009	−5.72%
	**Isoflurane**	1 mM *	0.2245 ± 0.0003	0.2218 ± 0.0005	−1.20%
		**5 mM**	0.2270 ± 0.0005	0.2162 ± 0.0006	−4.76%
	**Propofol**	1 µM *–10 µM *			No change
		**30 µM**	0.2251 ± 0.0009	0.2228 ± 0.0007	−1.02%
		**100 µM**	0.2240 ± 0.0006	0.2193 ± 0.0003	−2.10%
		**200 µM**	0.2279 ± 0.0025	0.2200 ± 0.0024	−3.47%
	**Ketamine/** **xylazine**	42 µM *			No change
		**210 µM**	0.2273 ± 0.0011	0.2251 ± 0.0017	−0.97%
**POPC/Chol**	**Sevoflurane**	0.25 mM *	0.1787 ± 0.0002	0.1770 ± 0.0005	−0.95%
		0.50 mM *	0.1793 ± 0.0002	0.1768 ± 0.0004	−1.39%
		**1.0 mM**	0.1785 ± 0.0003	0.1747 ± 0.0002	−2.13%
		**2.0 mM**	0.1797 ± 0.0002	0.1718 ± 0.0005	−4.40%
	**Isoflurane**	1 mM *	0.1773 ± 0.0006	0.1761 ± 0.0006	−0.68%
		**5 mM**	0.1766 ± 0.0001	0.1702 ± 0.0005	−3.62%
	**Propofol**	1 µM *–200 µM			No change
	**Ketamine/** **xylazine**	42 µM *	0.1770 ± 0.0007	0.1769 ± 0.0008	−0.06%
		**210 µM**	0.1761 ± 0.0018	0.1754 ± 0.0019	−0.39%
**5-Lipids**	**Sevoflurane**	0.25 mM *	0.1746 ± 0.0002	0.1735 ± 0.0006	−0.63%
		0.50 mM *	0.1750 ± 0.0009	0.1731 ± 0.0003	−1.08%
		**1.0 mM**	0.1744 ± 0.0005	0.1709 ± 0.0007	−2.01%
		**2.0 mM**	0.1760 ± 0.0006	0.1687 ± 0.0003	−4.15%
	**Isoflurane**	1 mM *	0.1749 ± 0.0008	0.1732 ± 0.0002	−0.97%
		**5 mM**	0.1748 ± 0.0008	0.1677 ± 0.0010	−4.06%
	**Propofol**	1 µM *–30 µM			No change
		**100 µM**	0.1757 ± 0.0010	0.1743 ± 0.0010	−0.80%
		**200 µM**	0.1757 ± 0.0006	0.1730 ± 0.0008	−1.54%
	**Ketamine/** **xylazine**	42 µM *			No change
		**210 µM**	0.1750 ± 0.0004	0.1744 ± 0.0004	−0.34%

**Table 3:** The percentage changes in TMA-DPH fluorescence anisotropy after adding anesthetics. The average measured value during the 10 min period before adding anesthetics is called the value “Before adding anesthetic”, while the average value between 8 and 15 min after adding anesthetics is called “After adding anesthetic”. Anesthetic concentrations with an asterisk (*) sign are considered to be clinical concentrations.

Drug Concentration	Before Adding Anesthetic	After Adding Anesthetic	Percentage Change
1 mM Isoflurane *	0.285177 ± 0.0011	0.2778 ± 0.0024	−2.59%
5 mM Isoflurane	0.285858 ± 0.0040	0.2746 ± 0.0052	−3.92%
0.25 mM Sevoflurane *	0.295056 ± 0.0029	0.2890 ± 0.0032	−2.03%
0.5 mM Sevoflurane *	0.287785 ± 0.0040	0.2798 ± 0.0035	−2.78%
1.0 mM Sevoflurane	0.292957 ± 0.0034	0.2845 ± 0.0036	−2.90%
2.0 mM Sevoflurane	0.284942 ± 0.0083	0.2743 ± 0.0090	−3.72%
174 mM Ethanol	0.302055 ± 0.0130	0.2981 ± 0.0124	−1.29%

## Data Availability

The original contributions presented in this study are included in the article. Further inquiries can be directed to the corresponding authors.
